# Analysis of the Generalization Ability of Defogging Algorithms on RICE Remote Sensing Images

**DOI:** 10.3390/s24144566

**Published:** 2024-07-14

**Authors:** Guisheng Miao, Zhongpeng Zhang, Zhanbei Wang

**Affiliations:** 1 School of Physics and Telecommunication Engineering, Shaanxi University of Technology, Hanzhong 723001, China; m_111_s@163.com (G.M.); wang-97221@163.com (Z.W.); 2 Trine Engineering Institute, Shaanxi University of Technology, Hanzhong 723001, China

**Keywords:** defogging algorithms, remotely sensed images, RICE dataset

## Abstract

This paper explores the generalization ability of defogging algorithms on RICE (A Remote Sensing Image Dataset for Cloud Removal) remotely sensed images. RICE is a dataset of remotely sensed images used for removing clouds, allowing the researcher to better evaluate the performance of defogging algorithms for cloud removal from remotely sensed images. In this paper, four classical defogging algorithms, including AOD-Net, FFA-Net, dark channel prior, and DehazeFormer, are selected and applied to the task of de-cloud in RICE remote sensing images. The performance of these algorithms on the RICE dataset is analyzed by comparing the experimental results, and their differences, advantages, and disadvantages in dealing with de-clouded remote sensing images are explored. The experimental results show that the four defogging algorithms are capable of performing well on uniform thin cloud images, but there is a color distortion and the performance is weak when it comes to inhomogeneous clouds as well as thick clouds. So, the generalization ability of the algorithms is weak when the defogging algorithms are applied to the problem of cloud removal. Finally, this paper proposes improvement ideas for the de-cloud problem of RICE remote sensing images and looks forward to possible future research directions.

## 1. Introduction

In today’s digital age, remote sensing technology has become one of the most important means of obtaining information about the Earth’s surface. Through remote sensing images, a variety of information about the Earth’s surface can be obtained, such as topography and geomorphology, vegetation cover, land use, and so on, providing important data support for resource management, environmental monitoring, urban planning, and other fields. However, due to the atmospheric conditions, remote sensing images are often affected by atmospheric disturbances such as haze, rain, fog, clouds, etc., resulting in image quality degradation and blurred information, which reduces their value and usability in practical applications [1].

In recent years, with the rapid development of artificial intelligence technologies such as deep learning, defogging algorithms have received widespread attention as an image enhancement technique [2]. These algorithms effectively remove disturbances such as haze and clouds by modeling and processing factors such as atmospheric scattering and lighting conditions, thus improving the visual quality of remote sensing images. However, most of the current defogging algorithms are designed for specific scenes or specific types of images. For example, the defogging algorithm proposed by Li et al. targets a variety of underwater images [3], the defogging algorithm proposed by Liu et al. targets images containing a large number of sky regions [4], and the algorithm proposed by Li et al. targets images of nighttime haze [5], while in practical applications the scenes and conditions of remote-sensing images are often diverse. Therefore, how to evaluate the generalization ability of defogging algorithms in different scenes has become an important topic in current research.

Among the many remote sensing image-defogging algorithms, algorithms such as AOD-Net (All-in-One Dehazing Network) [6], FFA-Net (Feature Fusion Attention Network for Single Image Dehazing) [7], dark channel a prior (Single Image Haze Removal Using Dark Channel Prior) [1], and DehazeFormer (Vision Transformers for Single Image Dehazing) [8] have achieved some success. These algorithms show different advantages in different scenarios; for example, AOD-Net and FFA-Net have better defogging effects in complex scenarios, the dark channel a prior algorithm has strong robustness, and DehazeFormer has high efficiency in dealing with large-scale data. In addition, some other defogging algorithms, such as GMAN (Single Image Dehazing with a Generic Model-Agnostic Convolutional Neural Network) [9], cycle dehaze (Cycle-Dehaze: Enhanced CycleGAN for Single Image Dehazing) [10], HyLoG-ViT (Complementary Feature Enhanced Network with Vision Transformer for Image Dehazing) [11], etc., have also achieved some results in the field of image defogging. However, there is a lack of in-depth comparisons and analyses of these algorithms for specific scenarios on specific datasets, especially for the de-clouding effect of remotely sensed images.

To fill this research gap, the RICE (A Remote Sensing Image Dataset for Cloud Removal) dataset is selected as the object of study in this paper and the performances of the AOD-Net, FFA-Net, dark channel a prior, and DehazeFormer algorithms on this dataset are comparatively analyzed.

The defogging algorithm based on the dark channel prior is an algorithm in the statistical sense. The dark channel defogging algorithm is an image processing technique based on the a prior theory of dark channels to recover a clear image from a hazy image. The core of the algorithm is to compute the dark channel of an image, which is the smallest value among multiple color channels that is used to represent the darkness of a pixel. The dark channel is then utilized to estimate the transmittance and atmospheric light values to remove the effects of haze. Dr. He summarized a large number of outdoor fog-free images and found that there are some pixels in the local region of the fog-free image. At least one of these pixels has a very low brightness value for at least one of the color channels (the region of the low brightness value excludes the sky region, for example). In the field of computer vision and computer graphics, a widely used formula to describe foggy images [12,13] is:

(1) $$I\left(x\right)=J\left(x\right)t\left(x\right)+A\left(1-t\left(x\right)\right)$$

where $I\left(x\right)$ denotes the foggy image, $J\left(x\right)$ is the fog-free image to be recovered, A is the global atmospheric light composition, and $t\left(x\right)$ is the transmittance. By using this equation, the goal is to compute the original fog-free image, the transmittance, and the estimation of the global atmospheric light composition from the available images.

The AOD-Net defogging algorithm is a deep learning-based approach to defogging that utilizes the powerful feature learning and mapping capabilities of neural networks. Conventional defogging methods typically rely on physical models and prior knowledge to estimate fog concentrations and attempt to remove haze. However, these methods usually require manual parameter tuning and have limited effectiveness in complex situations. Therefore, in recent years, the development of deep learning has provided new ways to solve these problems. Most deep learning methods for image restoration and enhancement fully incorporate end-to-end modeling: training models to regress clear images directly from corrupted ones. Examples include image denoising, deblurring, and super-resolution. In contrast, there is currently no end-to-end deep modeling for defogging that regresses clear images directly from blurred models and current models for defogging lack articulation with advanced vision tasks. By training a large number of fogged and clear image pairs, AOD-Net is able to learn how to convert fogged images into clear ones for efficient defogging. It adopts an end-to-end training approach, outputting clear images directly from the input fogged images, avoiding the tedious process of manually adjusting parameters. In addition, AOD-Net can be seamlessly embedded with other deep models to perform advanced tasks on foggy images [6].

The emergence of the FFA-Net defogging algorithm is mainly based on the development of deep learning techniques, the importance of image defogging, and the limitations of traditional methods. Image defogging is an important and challenging problem in the field of computer vision [14], and in recent years, with the rapid development and application of deep learning techniques, more and more image processing tasks have begun to adopt deep learning methods. These methods are usually able to train on large-scale datasets and achieve good performance on various vision tasks.

Image defogging, as an important image processing task, has received a lot of attention from both academia and industry. Clear images are crucial for many applications, such as unmanned vehicles, surveillance systems, satellite images, etc. Therefore, improving the effectiveness of image defogging has become an important research direction.

Traditional image defogging methods are usually based on physical models or a prior knowledge, which can improve image quality to a certain extent but often do not handle complex scenes well or provide high-quality results. As a result, researchers have begun to explore deep-learning-based approaches to address this problem. In the field of deep learning, feature fusion and attention mechanisms are widely used in a variety of tasks to enhance the network’s representation and improve task performance [15]. The development of these techniques provides new ideas and methods for image-defogging tasks. By combining feature fusion and attention mechanism, FFA-Net can effectively improve the effect of image defogging and provide a new solution to solve the image quality problem in practical applications.

DehazeFormer is an image-defogging method based on the Transformer architecture. The Transformer [16] model was originally proposed as a revolutionary model in the field of natural language processing but has since been shown to have broad applicability in other fields as well, including image processing. In image processing tasks, especially at the pixel level (e.g., image segmentation, denoising, etc.), the Transformer model has been shown to achieve impressive results. Therefore, it is a natural extension for Transformer-based algorithms to be used for tasks like image defogging.

The traditional defogging algorithm and CNN-based defogging algorithm methods have some limitations, such as the dependence on a prior and the inability to deal with high-frequency information. In recent years, with the rise of deep learning in the field of computer vision, image-defogging methods based on deep convolutional neural networks (CNNs) have made significant progress. However, recent studies have shown that the Transformer architecture outperforms most CNN architectures in advanced visual tasks, which has prompted researchers to start exploring the possibility of applying the Transformer in the field of image defogging [17]. DehazeFormer was inspired by the Swin Transformer [18], and through a series of improvements, it significantly outperforms traditional CNN-based methods, especially in processing high-frequency information.

The RICE dataset is a remote sensing image dataset for cloud removal that contains real remotely sensed cloudy images as well as the corresponding remotely sensed cloud-free images, with certain representativeness and practicability [19]. By comparing the experimental results, this paper will evaluate the advantages and disadvantages of each algorithm in terms of de-clouding effect, computational complexity, and applicable scenes and provide an important reference for the research and application of de-clouding algorithms for remote sensing images.

## 2. Materials and Methods

### 2.1. Dataset

**Figure 1:**
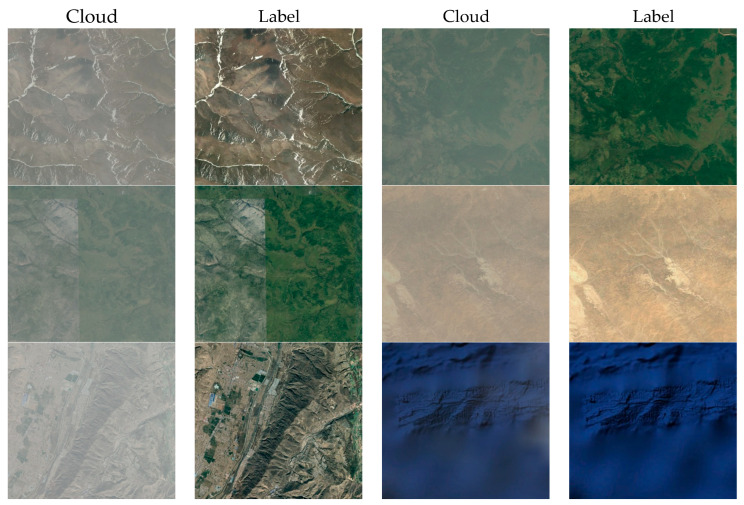
RICE-I dataset. The cloud column represents a blurred image with clouds, and the label column represents a clear image without clouds.

**Figure 2:**
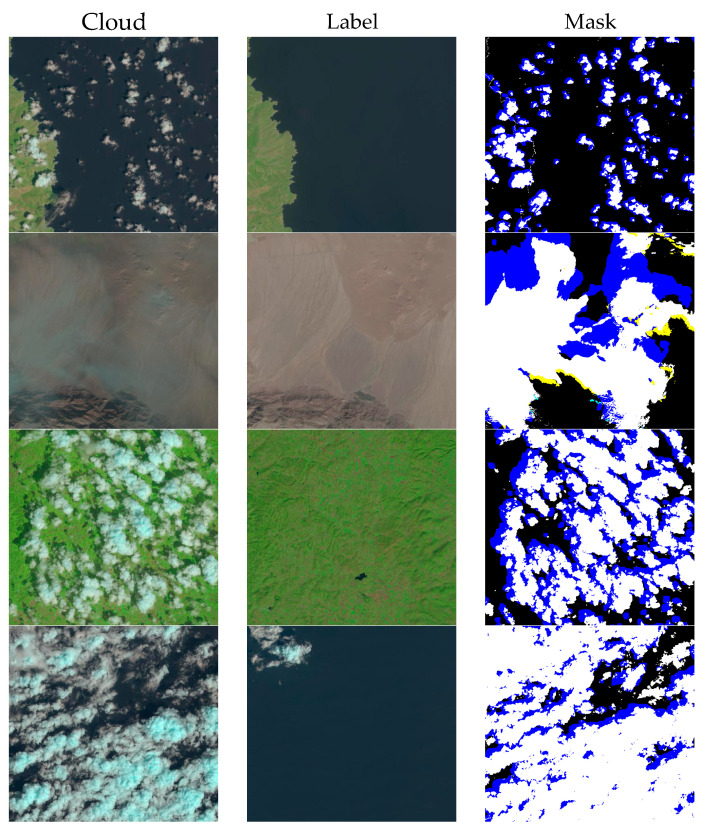
RICE-II dataset. The cloud column represents the blurred image with cloud, the label column represents the corresponding clear image without cloud, and the MASK column represents the corresponding mask image with cloud.

To verify the effectiveness of the algorithm, two benchmark datasets from RICE were used for the experiments:

1. RICE-I dataset. The RICE-I dataset contains 500 pairs of images, each pair of images has one image with clouds and one without clouds, which were obtained by setting the cloud display parameters on Google Earth. Subsequently, these images were cropped to a size of 512 × 512 pixels and did not contain overlapping regions, as shown in Figure 1
2. RICE-II dataset. The RICE-II dataset is derived from the Landsat 8 OLI_TIRS dataset and utilizes LandsatLook imagery from the US National Geological Survey Earth Explorer database. The dataset uses geo-referenced Landsat-Look imagery, both natural color (landsat8 OLI, bands 6, 5, 4) and high-quality imagery (8-bit files generated from Landsat Level-1 quality bands). The cloud images were produced by acquiring real remote sensing images in the same area at least every 15 days. All images were cropped to a size of 512 × 512 pixels and did not contain overlapping areas. The dataset contains 450 pairs of images, each of which is a reference image without clouds, an image with clouds, and its cloud MASK map. As shown in Figure 2.

### 2.2. Methods

#### 2.2.1. Dark Channel Prior

1. Theory

In the sky-free region of most fog-free images, there is at least one color channel in the pixels that has a very low luminance value. This lowest luminance value is almost equal to 0. Thus, for an image J, the dark channel is:

(2) $$J^{dark}\left(x\right)=\underset{c\in \left\{r,g,b\right\}}{min}\;\left(\underset{y\in \Omega \left(x\right)}{min}\;\left(J^{c}\left(y\right)\right)\right)$$

where $J^{c}$ is the color channel of J and $\Omega \left(x\right)$ is the local block centred at x. Observations show that the intensity of $J^{dark}$ is low and tends to zero except in the sky region if J is a fog-free outdoor image. The dark channel of J is referred to as $J^{dark}$ and the above statistical observation or knowledge is referred to as the dark channel prior.

The low brightness in the dark channel comes from several sources:

- Shadows
- Colored objects or surfaces
- Black objects or surfaces

2. Algorithm design

Assuming first that the atmospheric light component A is known, the defogging model Equation (1) can be reduced to the following equation by dividing both sides of the equation by A at the same time:

(3) $$\frac{I^{c}\left(x\right)}{A^{c}}=t\left(x\right)\frac{J^{c}\left(x\right)}{A^{c}}+1-t\left(x\right)$$

The superscript c denotes the RGB three-channel, further assuming that the transmittance $t\left(x\right)$ within each filter window is constant, denoted as $\bar{t}\left(x\right)$, and then calculating the dark channel simultaneously for both sides of the equation, i.e., making two minima operations, the following equation can be obtained:

(4) $$\underset{y\in \Omega \left(x\right)}{min}\;\left(\underset{c}{min}\;\frac{I^{c}\left(y\right)}{A^{c}}\right)=\bar{t}\left(x\right)\underset{y\in \Omega \left(x\right)}{min}\;\left(\underset{c}{min}\;\frac{J^{c}\left(y\right)}{A^{c}}\right)+1-\bar{t}\left(x\right)$$

Since $\bar{t}\left(x\right)$ is a constant, it is placed outside the minimum operation, and then, according to the dark-channel prior, $J^{dark}$ tends to zero, i.e.:

(5) $$J^{dark}\left(x\right)=\underset{y\in \Omega \left(x\right)}{min}\;\left(\underset{c}{min}\;J^{c}\left(y\right)\right)=0$$

Since $A^{c}$ is always positive, we can obtain:

(6) $$\underset{y\in \Omega \left(x\right)}{min}\;\left(\underset{c}{min}\;\frac{J^{c}\left(y\right)}{A^{c}}\right)=0$$

Substituting Equation (6) into Equation (4) yields an estimate of the transmittance.

(7) $$\bar{t}\left(x\right)=1-\underset{y\in \Omega \left(x\right)}{min}\;\left(\underset{c}{min}\;\frac{I^{c}\left(y\right)}{A^{c}}\right)$$

However, in reality, even on a clear day, the atmospheric light component is still present. Especially when it comes to looking at objects in the distance, you will notice that the fog is still there, and in addition, the presence of fog is a fundamental clue to human depth perception [20,21]. If the fog is completely removed, the image may look unnatural and the sense of depth may be lost, therefore, a constant parameter ω(0<ω<1) is introduced to control the degree of defogging. Inside the original paper the authors suggested that ω take 0.95.

(8) $$\bar{t}\left(x\right)=1-\omega \underset{y\in \Omega \left(x\right)}{min}\;\left(\underset{c}{min}\;\frac{I^{c}\left(y\right)}{A^{c}}\right)$$

Before, it was assumed that A is a known quantity, but the value of A can be approximated by finding the brightest points in the first 0.1% of the dark channels, i.e., the points with the smallest transmittance; for these points, go to the fog map to find the corresponding points and take the largest value of all the channels in them as an approximation to A. At this point, the transmittance $t\left(x\right)$, the atmospheric light component A, and the fog map $I\left(x\right)$, are all known and the fog-free map $J\left(x\right)$ can be solved for:

(9) $$J\left(x\right)=\frac{I\left(x\right)-A}{\max\left(t\left(x\right),t_{0}\right)}+A$$

where $t_{0}$ is a transmittance lower bound. Since the direct recovery when the transmittance $t\left(x\right)$ is close to zero from Equation (1), $J\left(x\right)$ $t\left(x\right)$ is also zero, which will lose the original image information and makes it easy to introduce noise, so a lower bound is set to retain a certain amount of fog in the place where the density of fog is very high. Inside the original paper, the authors suggested a value of 0.1 for $t_{0}$ because the image after defogging will generally look darker, so the exposure can be increased appropriately to obtain better results.

#### 2.2.2. AOD-Net

1. Theory

AOD-Net (All-In-One Dehazing Network) is a deep learning image-defogging algorithm based on a reformulated atmospheric scattering model that is based on the principle of using convolutional neural networks (CNN) and an end-to-end training approach. The core idea of the algorithm is to learn the mapping from foggy images to clear images by training an end-to-end neural network using the feature learning capability of deep learning. During the training of the network, supervised learning is performed using a large number of foggy and clear image pairs, enabling the network to learn the complex mapping relationship between the input image and the output image.

In AOD-Net, a convolutional neural network is first used to learn the features in a foggy image, which include the distribution of haze, the contrast of the image, and so on. Then, the network extracts and abstracts these features through a series of convolution and pooling operations and gradually converts them into a representation of a clear image. Finally, after processing in the output layer of the network, the difference between the generated image and the clear image is minimized, thus achieving the defogging effect.

This end-to-end training approach allows AOD-Net to automatically learn the process of image defogging without the need to manually adjust parameters or rely on prior knowledge. This enables AOD-Net to achieve good defogging results in a variety of different scenarios with strong generalization capabilities.

2. Algorithm design

Firstly, through the atmospheric scattering model in (1), both sides are simultaneously divided by $t\left(x\right)$ into Equation (10):

(10) $$J\left(x\right)=\frac{1}{t\left(x\right)}I\left(x\right)-A\frac{1}{t\left(x\right)}+A$$

Previous methods such as [2,22] estimated $t\left(x\right)$ and A, respectively, and obtained clear images by (10). Instead of directly minimizing the reconstruction error on $J\left(x\right)$, they go on to optimize $t\left(x\right)$. This indirect optimization leads to sub-optimal solutions, so the core idea here is to integrate the two parameters $t\left(x\right)$ and A into a single formula, $K\left(x\right)$ in (11), and to directly minimize the reconstruction error in the pixel domain of the image. Therefore, the formula in (10) is re-expressed as:

(11) $$\begin{array}{c} J\left(x\right)=K\left(x\right)I\left(x\right)-K\left(x\right)+b,where\\ K\left(x\right)=\frac{\frac{1}{t\left(x\right)}\left(I\left(x\right)-A\right)+\left(A-b\right)}{I\left(x\right)-1} \end{array}$$

In this way, $K\left(x\right)$ integrates $t\left(x\right)$ and A. Here, b is a constant bias with a default value of 1. As can be seen, $K\left(x\right)$ is also dependent on $I\left(x\right)$ and, therefore, an input-adaptive depth model needs to be built and the model needs to be trained by minimizing the error in reconstruction between its output $J\left(x\right)$ and the reconstruction error of the real clear image to train the model.

#### 2.2.3. FFA-Net

1. Theory

FFA-Net is a feature fusion attention network that fuses different types of information through a feature attention module and can process different types of information more flexibly. The structure of this network includes a shallow feature extraction part, a Group Architecture with multiple jump connections [7], a feature attention module, a reconstruction part, and a global residual learning structure.

The feature attention module consists of channel attention and pixel attention, which can provide additional flexibility to process different types of information. Channel attention focuses on the weighted information of different channel features and obtains the weights of different channels through global average pooling and convolutional layers. Feature fusion attention fuses features by splicing the feature maps output from Group Architecture in the channel direction and obtaining adaptive learning weights through the feature attention mechanism.

FFA-Net extracts features from the input haze-affected images using a deep convolutional neural network (CNN). These features usually contain semantic information, edge information, etc., of the image.

FFA-Net uses the technique of feature fusion to fuse features from different layers. The aim is to enhance the representational capabilities of the network so that it can better understand the structure and content of the image.

FFA-Net introduces an attention mechanism for dynamically adjusting how much attention the network pays to different features. This means that the network can automatically focus on feature regions that are more important to the image-defogging task when processing images, thus improving the defogging effect. The attention mechanism allows the network to pay more attention to important pixels or regions when processing different pixels, thus reducing the impact of irrelevant information.

Finally, after the feature fusion and attention mechanism, FFA-Net reconstructs the processed feature maps into a final defogged image. This process is usually achieved by operations such as deconvolution or upsampling.

2. Algorithm design

The algorithm design of FFA-Net consists of the following key elements: the feature attention module, basic block structure, feature fusion structure, and loss function.

**Figure 3:**
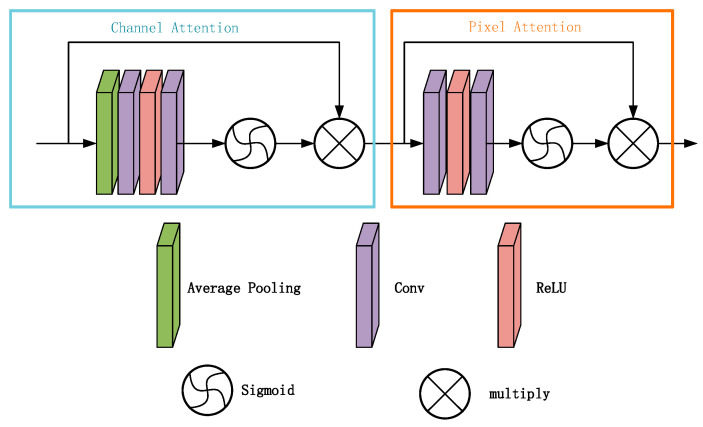
Feature attention module.

The feature attention module consists of a channel attention module and a pixel attention module (Figure 3). The channel attention mainly focuses on the weighting information of different channel features for image defogging, and the global spatial information related to the channel is put into the channel descriptor by using global average pooling.

(12) $$g_{c}=H_{p}\left(F_{c}\right)=\frac{1}{H\times W}\sum_{i=1}^{H}\;\sum_{j=1}^{W}\;X_{c}\left(i,j\right)$$

where $X_{c}\left(i,j\right)$ denotes the value of the cth channel $X_{c}$ at position $\left(i,j\right)$ and $H_{p}$ is the global pooling function. To obtain the weights of the different channels, the features are passed through two convolutional layers and sigmoid [23] and ReLu [24] activation functions.

(13) $$CA_{c}=\sigma \left(Conv\left(\delta \left(Conv\left(g_{c}\right)\right)\right)\right)$$

where σ is the sigmoid function and δ is the ReLu function. Finally, the input $F_{C}$ is multiplied element-by-element with the weights of the channel $CA_{c}$.

(14) $$F_{C}=CA_{c}⊗F_{c}$$

Pixel attention is similar to channel attention in that we directly feed the input F (the output of the CA) into two convolutional layers with ReLu and sigmoid activation functions.

**Figure 4:**
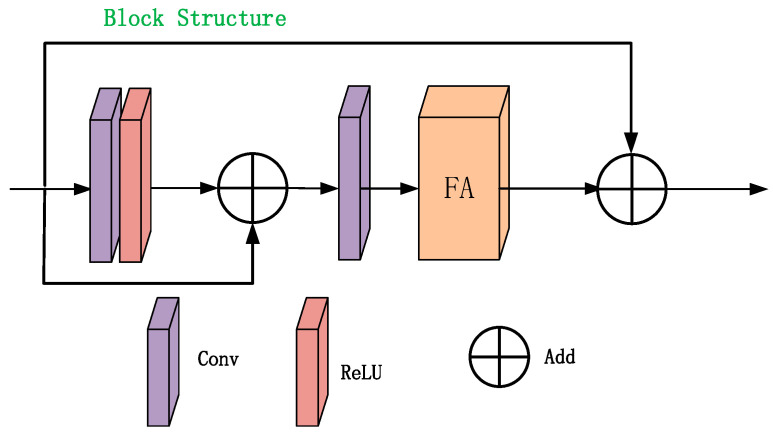
Basic block structure.

The basic block structure consists of local residual learning and feature attention (FA) modules (Figure 4).

The feature fusion architecture first stitches all the feature maps output by multiple Group Architectures in the channel direction and then perform feature fusion by the adaptive learning weights obtained from the Feature Attention (FA) mechanism.

The loss function uses a simple L1 loss function, and the network is trained by optimizing the L1 loss function to improve performance.

(15) $$L\left(\Theta \right)=\frac{1}{N}\sum_{i=1}^{N}\;∥I_{gt}^{i}-FFA\left(I_{haze}^{i}\right)∥$$

where Θ denotes the parameters of the FFA-Net network, $I_{gt}$ denotes the ground truth, and $I_{\mathrm{haze}}$ denotes the input.

#### 2.2.4. DehazeFormer

1. Theory

The principle of the DehazeFormer algorithm is an image-defogging method based on Transformer architecture. Inspired by Swin Transformer, DehazeFormer surpasses the traditional CNN-based methods through a series of improvements.

DehazeFormer has been designed with some key improvements, including removing the LayerNorm [25], which is commonly used in Vision Transformer [17], and proposing RescaleNorm in place of LayerNorm to better preserve the correlation between features.

For the choice of activation function, the original authors of DehazeFormer proposed SoftReLU, which performs better in the image-defogging task compared to SiLU/Swish [26] and GELU [27]. For window segmentation in Transformer, DehazeFormer proposes an offset window segmentation scheme based on reflection filling and cropping. In terms of spatial information aggregation, DehazeFormer introduces the scheme of W-MHSA combined with parallel convolution to improve the performance of the network by aggregating information within a window while using convolution to aggregate information within a neighborhood. A prior soft reconstruction module, which outperforms global residual learning and SKNet-based multi-scale feature map fusion module [28], is also introduced to replace the cascade fusion.

2. Algorithm design

The algorithm design of DehazeFormer includes the following key elements: a multi-scale feature map fusion module and a prior soft reconstruction module, normalization substitution, activation function selection, and W-MHSA combined with parallel convolution.

Firstly in the SKNet-based multi-scale feature map fusion module, to first assume that the two features are mapped as $x_{1}$ and $x_{2}$, using a linear layer f (·) to project $x_{1}$ to ${\hat{x}}_{1}$, we use global average pooling GAP, $F_{MLP}$, the softmax function, and the splitting operation to obtain the fusion weights:

(16) $$\{a_{1},a_{2}\}=\mathrm{Split}\;(\mathrm{Softmax}\left(F_{MLP}\left(\mathrm{GAP}\left({\hat{x}}_{1}+x_{2}\right)\right)\right)$$

Use weights $\left\{a_{1},a_{2}\right\}$ to fuse ${\hat{x}}_{1}$ and $x_{2}$ with additional short residuals via $\begin{array}{c} y=a_{1}{\hat{x}}_{1}+a_{2}x_{2}+x_{2} \end{array}$.

The a-prior-based soft reconstruction module is a reconstruction of Equation (1):

(17) $$\begin{array}{c} J\left(x\right)=K\left(x\right)I\left(x\right)+B\left(x\right)+I\left(x\right) \end{array}$$

where $\begin{array}{c} K(x)=1/t(x)-1 \end{array},\;B(x)=-\left(1/t(x)-1\right)A$.

RescaleNorm is an improvement on LayerNorm; the normalization process of LayerNorm can be represented as:

(18) $${\hat{x}}_{i}=\frac{x_{i}-\mu_{i}}{\sigma_{i}}\cdot \gamma_{i}+\beta_{i}$$

Here, μ and σ denote the mean and standard deviation, γ and β are the learned scale factor and bias, and $\begin{array}{c} i=(i_{b},i_{n},i_{c}) \end{array}$ denotes the index. Firstly, we first obtain $\begin{array}{c} \mu ,\sigma \in R^{b\times 1\times 1} \end{array}$ and normalize the input feature x to $\hat{x}$ by Equation (18); then, we process $\hat{x}$ to obtain the output $\hat{y}$ by using the F(·) module. We use two linear layers with weights $W_{\gamma },W_{\beta }\in R^{1\times c}$ and bias $B_{\gamma },B_{\beta }\in {\tilde{R}}^{1\times 1\times c}$, which is passed through $\left\{\gamma ,\left.\beta \right\}\right.=\sigma W_{\gamma }+B_{\gamma },\mu W_{\beta }+B_{\beta }$ to change the values of µ and σ.

(19) $$y=F\left(\frac{x-\mu }{\sigma }\cdot \gamma +\beta \right)\cdot \left(\sigma W_{\gamma }+B_{\gamma }\right)+\left(\mu W_{\beta }+B_{\beta }\right)$$

where $\gamma ,\beta \in R^{b\times 1\times c}$.

The algorithm design of SoftReLU is an approximation of ReLU [29].

(20) $$\mathrm{SoftReLU}\left(x\right)=\frac{x+\sqrt{x^{2}+\alpha^{2}}-\alpha }{2}$$

where α is a shape parameter and SoftReLU is equivalent to ReLU when α = 0 is set.

W-MHSA combined with parallel convolution is used to introduce parallel convolution based on Multihead Self-Attention Mechanism (MHSA) to improve the network’s ability to extract features at different scales and the effect of defogging. In W-MHSA combined parallel convolution, the MHSA is firstly used for spatial information aggregation and the attention mechanism is applied to the information aggregation within the window to achieve the attention and integration of local information. In contrast to the spatial information aggregation approach of MHSA, W-MHSA introduces an additional convolution operation to aggregate the features but does not consider the window division to achieve the aggregation of neighborhood information. Thus, spatial information aggregation can be expressed as:

(21) $$\;\mathrm{Aggregation}\;\left(Q,K,V\right)=Softmax\left(QK^{T}/\sqrt{d}+B\right)V+Conv\left(\hat{V}\right)$$

where $\hat{V}\in R^{b\times h\times w\times c}$.

Specifically, the computational process of W-MHSA combined with parallel convolution is as follows: firstly, the convolution operation is performed on the input features V before window division to obtain the convolved features; then, the MHSA mechanism is used to perform the attentional weighted aggregation of the information inside the window; finally, the weighted information obtained from MHSA is added with the convolved features to realize the fusion of the in-window and out-of-window information.

### 2.3. Experimental Setting

#### 2.3.1. Dataset

The RICE remote sensing image dataset was selected as the experimental dataset. The RICE dataset contains a large number of remote sensing images covering different scenarios and conditions. So, a portion of images from this dataset was randomly selected as experimental samples.

#### 2.3.2. Defogging Algorithm

Four different defogging algorithms were selected for comparison, namely:

- Single Image Haze Removal Using Dark Channel Prior (DCP)
- All-in-One Dehazing Network (AOD-Net)
- Feature Fusion Attention Network for Single Image Dehazing (FFA-Net)
- Vision Transformers for Single Image Dehazing (DehazeFormer)

#### 2.3.3. Experimental Step

Since the dark channel prior algorithm does not use a neural network, it is like a physical filter, so we do not have to perform any training operation on the dark channel prior algorithm model. However, the AOD-Net, FFA-Net, and DehazeFormer algorithms all use neural networks and attention mechanisms and there are original authors and trained pre-training models under each algorithm. So, in order to maintain the consistency and fairness of the experiments, we will first use the three algorithms, AOD-Net, FFA-Net, and DehazeFormer. The original trained pre-trained models are validated on the RICE dataset.

Secondly, we also fine-tuned the parameters on the three algorithmic models of AOD-Net, FFA-Net, and DehazeFormer according to the characteristics of the dataset and then trained using the RICE dataset to obtain the training model under each algorithm, with guided filtering added on top of the dark channel a prior algorithm, which better preserves the edge features after processing.

Finally, we compare the models obtained from the three algorithms (AOD-Net, FFA-Net, and DehazeFormer) trained on the RICE dataset with the pre-trained models provided by the original authors and select the models that are good within the two to be used for testing.

#### 2.3.4. Evaluation Indicators

Two evaluation metrics based on image clarity were chosen to assess the de-cloud effect of different algorithms, PSNR (peak signal-to-noise ratio) [30] and SSIM (Structural Similarity Index) [31], which are used to measure the degree of similarity between the de-clouded image and the original image. PSNR is often used as a measure of the quality of signal reconstruction in areas such as image compression, where a larger PSNR value indicates a better-quality image, which is often defined simply by the Mean Square Error (MSE). For two m × n monochrome images I and K, if one is a noise approximation of the other, then their mean square error is defined as:

(22) $$MSE=\frac{1}{mn}\sum_{i=0}^{m-1}\;\sum_{j=0}^{n-1}\;[I(i,j)-K(i,j){]}^{2}$$

The peak signal-to-noise ratio is defined as:

(23) $$PSNR=10\cdot \log_{10}\left(\frac{MAX_{I}^{2}}{MSE}\right)=20\cdot \log_{10}\left(\frac{MAX_{I}}{\sqrt{MSE}}\right)$$

where *MAX_I_* is the maximum value that represents the color of an image point, which is 255 if each sample point is represented by 8 bits.

SSIM is a metric used to measure the degree of similarity between two digital images, the larger the value of SSIM, the higher the structural similarity between the two images. When one of the two images is a distortion-free image and the other is a distorted image, the structural similarity of the two can be seen as an image quality measure of the distorted image. Given two images X and Y, the structural similarity of the two is defined as:

(24) $$\begin{array}{c} SSIM(x,y)=[l(x,y){]}^{\alpha }[c(x,y){]}^{\beta }[s(x,y){]}^{\gamma },\\ l(x,y)=\frac{2\mu_{x}\mu_{y}+C_{1}}{\mu_{x}^{2}+\mu_{y}^{2}+C_{1}},c(x,y)=\frac{2\sigma_{x}\sigma_{y}+C_{2}}{\sigma_{x}^{2}+\sigma_{y}^{2}+C_{2}},s(x,y)=\frac{\sigma_{xy}+C_{3}}{\sigma_{x}\sigma_{y}+C_{3}} \end{array}$$

where l(x,y) compares the luminance of X and Y, c(x,y) compares the contrast of X and Y, and s(x,y) compares the structure of X and Y, with α > 0, β > 0, and γ > 0 as the adjusting l(x,y), c(x,y), s(x,y) parameters of relative importance and $\mu_{x}$ and $\mu_{y}$ and $\sigma_{x}$ and $\sigma_{y}$ as the means and standard deviations of X and Y, respectively. $\sigma_{x}\sigma_{y}$ are the covariances of X and Y. $C_{1}$, $C_{2}$, and $C_{3}$ are constants.

Since remote sensing images have strong structural information and SSIM can better consider the structural similarity of the images, the ratio of PSNR to SSIM is set to be 3:7 and the value of the SSIM index is more valued in the evaluation.

#### 2.3.5. Experimental Procedures

- Randomly selected a batch of remote sensing images from the RICE dataset as experimental samples.
- De-cloud selected images using the best model of each of the four algorithms.
- Evaluate the de-clouded images and record the values of the evaluation metrics.
- Analyze and compare the experimental results of different algorithms to explore their advantages, disadvantages, and applicable scenarios in terms of cloud removal effects.

## 3. Results

### 3.1. Selection of Pre-Trained Models

Among the four algorithms, because the original author of the DehazeFormer algorithm provides pre-training models for multiple scenarios as well as multiple datasets, to ensure the best effect of this algorithm on the RICE dataset, all the pre-training models of this algorithm were validated on this dataset in the preparation stage of the experiments. Because the RICE dataset contains two benchmark datasets and there are only 950 images in total, which constitutes fewer data, and many of the images inside the two benchmark datasets are from the same scene, for validation on the two benchmark datasets, the total number of images validated on each dataset was taken to be 100 images, which were randomly selected inside the respective dataset. Each pre-trained model under the algorithm was then evaluated and the pre-trained model with the best evaluation results under the algorithm was selected.

Each pre-training model was evaluated using the same metrics, i.e., PSNR and SSIM. At the end of the validation of each pre-training model, the PSNR and SSIM were computed for each image processing result and, finally, the PSNR and SSIM were averaged so that the PSNR and SSIM values of each pre-training model were obtained. Then, when evaluating the pre-trained models, the PSNR and SSIM were calculated according to the ratio 3:7. By calculating this ratio, the model with the largest score is selected as the pre-trained model under this algorithm for the corresponding dataset.

Since the DehazeFormer algorithm is trained and tested on the RESIDE [32] dataset and the RS-Haze dataset created by the original author, the original author also divided the RESIDE dataset into two different experimental settings according to different data distributions, namely RESIDE-Full and RESIDE-6K, where RESIDE-Full is further divided into indoor and outdoor. Therefore, there are four categories of pre-trained models under the DehazeFormer algorithm, namely indoor, outdoor, RESIDE-6k, and RS-haze. In the RESIDE-Full experimental setup, the indoor scenario mainly measures the model’s ability to process high-frequency information, while the outdoor scenario mainly measures the model’s convergence speed. In the RESIDE-6K experimental setup, the stability of the model and the ability to extract low-frequency information are mainly measured. In the RS-Haze dataset, the ability of the network to extract semantic features is mainly measured. There are trained pre-trained models under each category. The suffixes of the models, t, s, b, and m, represent tiny, small, basic, and medium, respectively. Under the four categories of the DehazeFormer algorithm, the PSNR and SSIM values of all pre-trained models verified on the RICE-I dataset are shown in Table 1, Table 2, Table 3 and Table 4, respectively.

From the data in the above four tables, it is known that the dehazeformer-m pre-trained model under the outdoor category is the most effective, so the dehazeformer-m pre-trained model under the outdoor category is selected as the pre-trained model under the RICE-I dataset under the DehazeFormer algorithm.

Under the four categories of the DehazeFormer algorithm, the PSNR and SSIM values of all pre-trained models verified on the RICE-II dataset are shown in Table 5, Table 6, Table 7 and Table 8, respectively.

From the data in Table 5, Table 6, Table 7 and Table 8, it is known that the dehazeformer-b pre-trained model under the RS-haze category is the best, so the dehazeformer-b pre-trained model under the RS-haze category is selected as the pre-trained model under the RICE-II dataset under the DehazeFormer algorithm.

### 3.2. Training Models

In this section, the main focus is on training the three algorithms, AOD-Net, FFA-Net, and DehazeFormer, and we fine-tuned the raw parameters of the three algorithms for better training. During the training process, we recorded the loss function values obtained from the model training, and when the loss function value reached the lowest, we stored the model parameters at that time as the best model under the training of that algorithm. Based on the total number of images, we selected 200 pairs of images for training the model on the RICE-I dataset and 200 pairs of images for training the model on the RICE-II dataset. Because of the small size of the dataset, the 200 pairs of data we selected for training the model in the DehazeFormer algorithm contained data from the 100 pairs that had been used previously in the pre-training model selection for DehazeFormer. This was for the sake of design rationality as well as to avoid overlap between the trained data and the subsequent test data. The models obtained by the three algorithms trained on the RICE-I dataset are shown in Table 9, and the models trained on the RICE-II dataset are shown in Table 10. Here, we name the training model for each algorithm to facilitate subsequent use.

### 3.3. Comparison of Trained and Pre-Trained Models

On RICE-I and RICE-II, we each selected 100 pairs of data that did not include the data we used to train the models. The results of the comparison between the original pre-trained models of the three algorithms and the models trained on the RICE dataset are shown in Table 11. The model with the highest score for both was selected as the final model to be tested.

Table 11 shows that on the RICE-I dataset, the pre-trained model of AOD-Net performs better than the trained model and the pre-trained model of FFA-Net also performs better than the trained model. Still, the trained model of DehazeFormer performs better than the pre-trained model. On the RICE-II dataset, the trained model of AOD-Net performs better than the pre-trained model, the pre-trained model of FFA-Net still performs better than the trained model, and the trained model of DehazeFormer still performs better than the pre-trained model.

**Figure 5:**
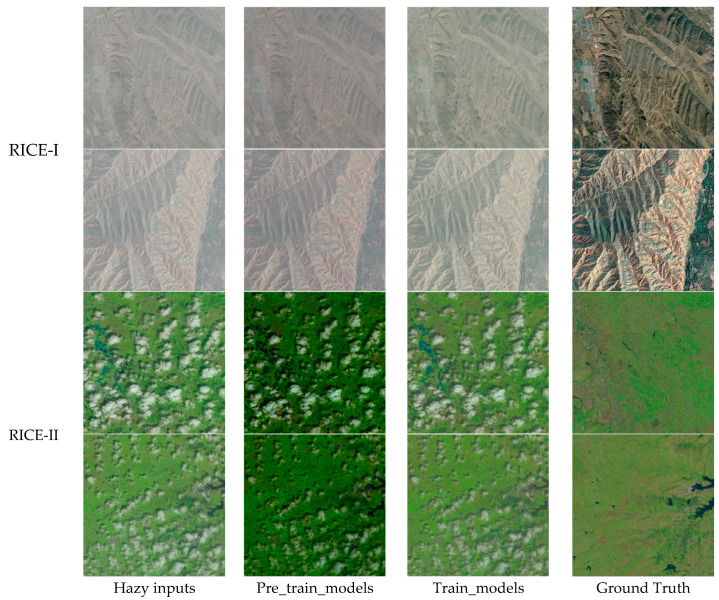
Comparison of AOD-Net’s pre-trained and trained models on the RICE-I and RICE-II datasets. The first column represents the original input image with fog, the second column represents the image processed using the pre-trained model, the third column represents the image processed using the trained model, and the fourth column represents the original clear image without fog. The first two rows represent the images inside the RICE-I dataset, and the last two rows represent the images inside the RICE-II dataset.

**Figure 6:**
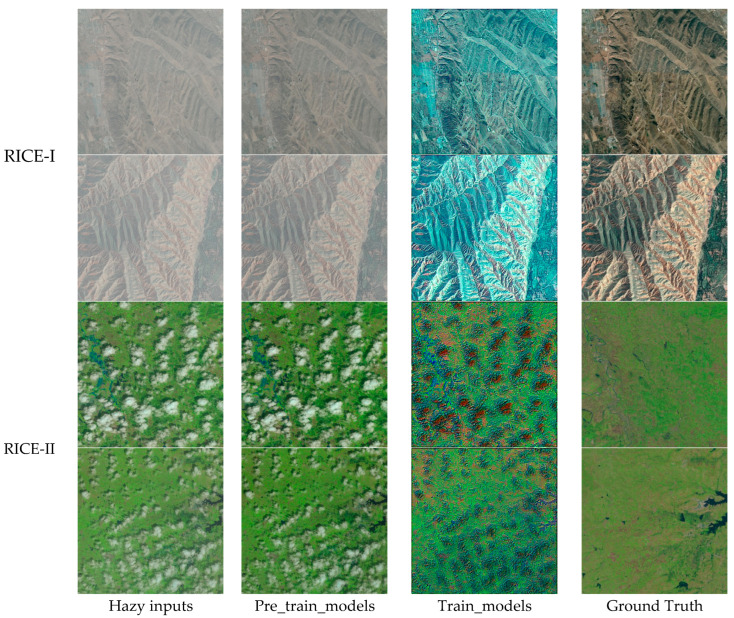
Comparison of FFA-Net’s pre-trained and trained models on the RICE-I and RICE-II datasets.

**Figure 7:**
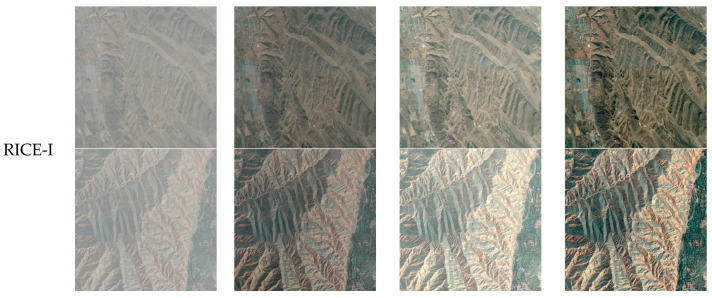

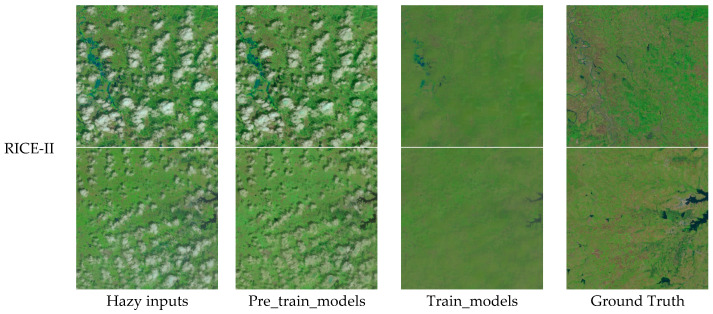
Comparison of DehazeFormer’s pre-trained and trained models on the RICE-I and RICE-II datasets.

Figure 5, Figure 6 and Figure 7 show the processing results of each algorithm’s pre-trained and trained models on the RICE-I and RICE-II datasets. It can be seen that the results of AOD-Net and FFA-Net processing using trained models are also not good and that the trained model of DehazeFormer performs better. Therefore, the DehazeFormer algorithm is more capable of generalization compared to the AOD-Net and FFA-Net algorithms.

### 3.4. Model Testing

In this stage of model testing, the pre-training model and the training model under each algorithm will be used as the test model after comparing the best results. To avoid overlap between the data used in testing and the data used in pre-training model selection, the datasets used in testing are excluded by combining the datasets that have been used in the pre-training model selection with those that the training model has used. Because the RICE-I dataset contains a total of 500 pairs of data and the RICE-II dataset includes a total of 450 pairs of data, after removing the 200 pairs of data used in training model selection and the 100 pairs of data used in the model comparison, there are still 200 pairs of data in the RICE-I dataset and 150 pairs of data in the RICE-II dataset. Therefore, for testing, we selected the remaining 200 pairs of images for testing in RICE-I and the remaining 150 pairs of images in RICE-II. Each algorithm is compared on two benchmark datasets, the results of the comparison on the RICE-I dataset are shown in Table 12, and the results of the comparison on the RICE-II dataset are shown in Table 13.

**Figure 8:**
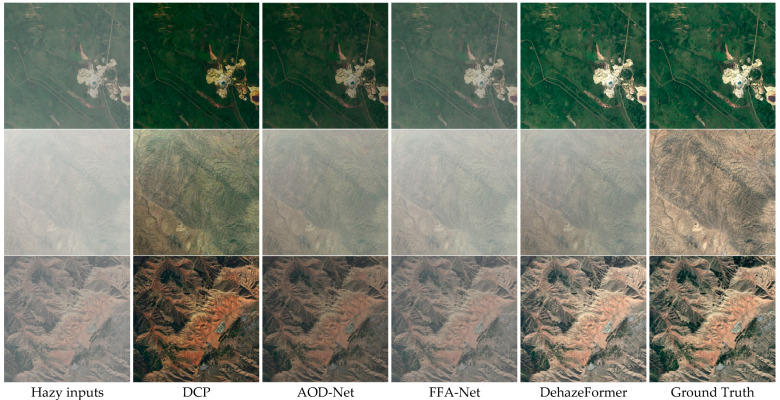
Comparison of RICE-I remote sensing images on four algorithms, DCP, AOD-Net, FFA-Net, and DehazeFormer. The first column represents the original input fogged image, the last column represents the original clear image without fog, and the middle four columns represent the images after being processed by each algorithm.

As can be seen from Table 12, the DehazeFormer algorithm has the best performance, with the highest PSNR and SSIM values under the RICE-I dataset. The PSNR value of DCP is the lowest, but as seen in Figure 8, the DCP algorithm is also effective in removing clouds. This is because PSNR mainly measures the overall brightness and noise level of the image. For the processed image, although the visual effect is improved, if the brightness and contrast adjustments are large, it may lead to a decrease in PSNR. In Figure 8, the image processed by the DCP algorithm is compared to the images processed by the other three algorithms when compared to the clear image. The image processed by the DCP algorithm suffers from severe color and contrast distortion and, as a result, the DCP has the lowest PSNR value.

As can be seen from Table 13, the DehazeFormer algorithm still performs best with the RICE-II dataset.

## 4. Discussion

In the RICE-I dataset, most of the images are fuzzy images shrouded by some uniform thin clouds and there are also some images with relatively thick clouds. Here, three images with different cloud concentrations are selected to evaluate the network’s performance regarding de-clouding clouds. For thin clouds, all four algorithms perform well in cloud removal; however, in restoring a clear image, three of the algorithms (except for the DehazeFormer algorithm) do not handle the color and details very well (see the first and third rows of Figure 8).

The DCP algorithm performs better when the cloud layer is relatively thick and the concentration is relatively high, where it can remove most of the clouds, but when recovering a clear image, the DCP-algorithm-processed image has distortion and poor color reproduction compared to the original clear image. The other three algorithms are not effective in removing most of the clouds when the cloud concentration is relatively high. See the second row of Figure 8.

The reason for the above situation may be because under uniform thin clouds the ground scene is relatively clear, the color and texture changes are small, and the effect of occlusion is relatively small, and these characteristics are conducive to the processing of the image by the defogging algorithm and thus obtaining a better de-clouding effect. The AOD-Net network was originally designed for defogging through a simple end-to-end architecture and may not have been tuned for dealing with a specific type of noise like clouds, which can lead to color distortion. Although FFA-Net introduces feature fusion and attention mechanisms, these mechanisms are mainly used to enhance feature extraction and are not specifically optimized for clouds, a type of noise that can also cause color distortion. DehazeFormer is based on Transformer’s architecture, which excels at capturing global information and reduces color distortion through a self-attention mechanism that allows for more precise adjustment of features.

When clouds are uniformly thicker, thick clouds scatter and absorb light more strongly, making the loss of contrast and detail in the image more severe. This places higher demands on the defogging (cloud removal) algorithm, which requires stronger feature extraction and recovery capabilities. Thick clouds cause many details in the image to be completely obscured, so relying solely on existing feature extraction techniques may not be sufficient to recover these details and hence the three algorithms AOD-Net, FFA-Net, and DehazeFormer do not perform well.

The DCP algorithm in this paper, on the other hand, employs guided filtering using the grayscale map of the image to be processed as the guide map, which better preserves the detailed features of the edges and reduces the blurring and artifacts that can be caused by the cloud removal process. In removing uniform thin clouds, which usually do not completely obscure the background information, the dark channel prior can effectively recognize and remove the interference caused by these thin clouds and restore the contrast of the image. Although thicker clouds are more complex, they still do not completely obscure the background information. By extracting the darkest channel information in the image, the DCP can estimate the atmospheric light and the transmission rate very well, thus effectively removing the effects brought by the clouds and thus removing the clouds with relatively good results. Also, because the luminance variations in clouds are different from haze, this may lead to the failure of the a prior assumptions of the dark channels, especially in thick cloud regions, which may lead to inaccurate color restoration. At the same time, the inconsistent effect of cloud cover on different color channels may lead to poor restoration of certain color channels, which may result in overall image color distortion.

**Figure 9:**
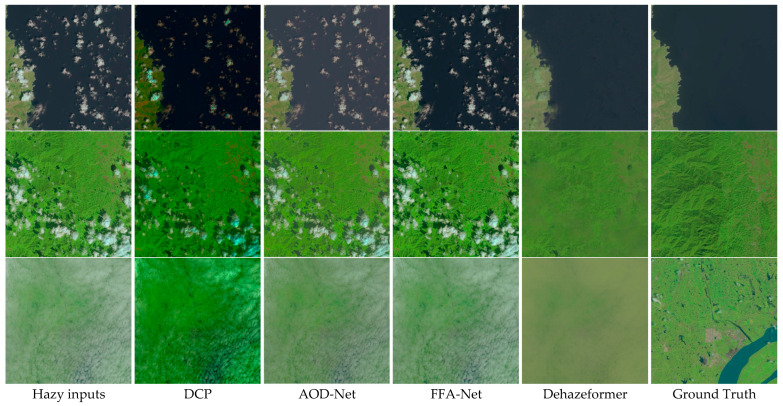
Comparison of RICE-II remote sensing images on four algorithms, DCP, AOD-Net, FFA-Net, and DehazeFormer.

In the RICE-II dataset, there are some fuzzy images shrouded by some irregularly shaped and non-uniform clouds. Again, three different images are selected, and it can be seen from Figure 9 that the three algorithms, DCP, AOD-Net, and FFA-Net, perform poorly on the RICE-II dataset. The DehazeFormer algorithm removes clouds but does not restore a clear image in its entirety.

The AOD-Net, FFA-Net, and DehazeFormer algorithms may produce images like this due to the following reasons: First of all, in the case of uneven cloud shapes, there may be a large cloud mass obscuring the ground scene, resulting in partial or total occlusion of the ground scene. General defogging algorithms find it difficult to deal with this kind of occlusion problem efficiently because they usually assume that the entire image scene is affected by the same degree of haze and ignore the localized occlusion. Secondly, in the case of thicker clouds, the clouds may reflect and scatter light more strongly, resulting in reduced visibility of the ground scene, and the defogging algorithms may not be able to accurately estimate the thickness and optical properties of the clouds to correctly restore the clarity of the ground scene. Finally, due to the uneven cloud shape and thickness inhomogeneity, some areas may be affected by stronger occlusions and reflections, resulting in localized distortions of the color and texture of ground scenes, and the defogging algorithms may also not be able to accurately recover these localized details, leading to poor results.

The reason for the poor performance results of the DCP defogging algorithm on image defogging may be that the principle of the DCP algorithm is based on the dark channel a prior assumption that most of the localized regions in a natural image are relatively dark in at least one color channel. This assumption applies to image blurring due to atmospheric scattering but not to clouds. Clouds may have higher reflectivity and more complex color distributions that do not have a dark channel prior, making it difficult for the defogging algorithm to accurately estimate a clear image.

The performance of these four defogging algorithms on the RICE dataset varies through model testing and analysis. For the RICE-I dataset, all of the algorithms perform better when dealing with uniform thin clouds, but the DCP and DehazeFormer algorithms perform better when dealing with relatively uniform thick clouds. For the RICE-II dataset, which has irregular shapes, unevenly distributed clouds, and thick clouds, the DehazeFormer algorithm has the best de-clouding ability compared to the other three algorithms. The computational complexity of each algorithm is different, so the speed of processing images is different, and each algorithm is tested using Windows with an RTX 4060 graphics card. Table 14 shows the number of parameters and FLOPs of each algorithm test model in this paper by taking the number of parameters and FLOPs index as a measure of the computational complexity because the DCP algorithm in this paper does not use neural networks and attention mechanisms so is not compared in the table; however, the DCP algorithm is designed to have a low complexity, processing an average of 10 images a second with an image size of 512 × 512 using an RTX 4060 graphics card and Windows 11.

As we can see from the data in the table, DehazeFormer has a lot of parameters due to the inclusion of a large number of attention mechanisms, low FLOPs, and a fast processing speed, so it is more suitable for the real-time processing of large datasets and its model performs more powerfully, adapts to more complex tasks, and has a better ability to learn and generalize. However, attention should be paid to preventing overfitting. The AOD-Net and FFA-Net algorithms have high FLOPs because they use convolution a lot, so the processing speed is slower.

In summary, when we are removing thin cloud pictures with a relatively uniform distribution, we can choose the four defogging algorithms in this paper. If the computer’s arithmetic power is relatively low, then we can choose the DCP algorithm. If the arithmetic power is relatively high and you want to process in real time you can choose the DehazeFormer algorithm. The AOD-Net and FFA-Net algorithms can be selected when dealing with a single dataset and real-time processing is not required. When removing thick clouds and images covered by uneven cloud cover, the above four algorithms are no longer applicable and we should improve the defogging algorithm according to the characteristics of the cloud cover of remote sensing images.

## 5. Conclusions

In this paper, the generalization ability of the current mainstream four defogging algorithms in the direction of remote sensing defogging is compared and analyzed, and after the experiments and the experimental data, it is concluded that the four defogging algorithms in this paper have a weak generalization ability in the direction of remote sensing defogging on the RICE remote sensing image dataset and cannot be applied to remote sensing image defogging.

There are several ideas for improvement in the problem of de-clouding remote sensing images:

- Design of specialized algorithms for different types of clouds based on the characteristics of different types of clouds; targeted cloud removal algorithms can be designed to improve the algorithm’s effectiveness in dealing with different types of clouds.
- Combining multiple sensor data for cloud identification and removal, where different types of sensors can provide complementary information to help accurately identify and remove clouds, thus improving the accuracy and robustness of the cloud removal algorithm.
- End-to-end cloud removal from remote sensing images using deep learning methods that are capable of learning complex image features and patterns to improve the generalization ability and effectiveness of cloud removal algorithms.
- Addition of ground-truth and synthetic datasets, including various types and densities of cloud cover scenarios. With richer datasets, the de-clouding algorithms can be better trained and evaluated, improving their generalization and applicability.

Future research on this subject will probably be in the following areas:

- Multimodal data fusion: further research on how to fuse multiple sensor data to improve the accuracy and effectiveness of cloud identification and removal.
- Optimization for specific application scenarios: optimize the de-clouding algorithm for specific remote sensing application scenarios, such as agricultural monitoring, urban planning, etc., to improve its applicability and effectiveness in specific scenarios.
- Improvement of real-time capability and efficiency: study how to improve the real-time capability and efficiency of the de-clouding algorithm so that it can be effectively applied in large-scale remote sensing image processing.
- Processing in conjunction with spatial and temporal information: combining spatial and temporal information to dynamically monitor and process remotely sensed images to better understand and respond to cloud cover changes at different temporal and spatial scales.

## Figures and Tables

**Table 1:** PSNR and SSIM values were obtained from the validation of pre-trained models under the indoor category on the RICE-I dataset.

Model	PSNR	SSIM	psnr×0.3+ssim×0.7
dehazeformer-b	18.15	0.834	6.03
dehazeformer-m	17.09	0.773	5.67
dehazeformer-s	17.52	0.815	5.83
dehazeformer-t	18.30	0.848	6.08

Note: The number highlighted in each column of the table is the maximum value for that column.

**Table 2:** PSNR and SSIM values were obtained from the validation of pre-trained models under the outdoor category on the RICE-I dataset.

Model	PSNR	SSIM	psnr×0.3+ssim×0.7
dehazeformer-b	20.75	0.885	6.84
dehazeformer-m	21.05	0.886	6.93
dehazeformer-s	20.81	0.882	6.86
dehazeformer-t	20.82	0.882	6.86

**Table 3:** PSNR and SSIM values were obtained from the validation of pre-trained models under the RESIDE-6K category on the RICE-I dataset.

Model	PSNR	SSIM	psnr×0.3+ssim×0.7
dehazeformer-b	20.70	0.886	6.83
dehazeformer-m	20.67	0.873	6.81
dehazeformer-s	20.86	0.868	6.86
dehazeformer-t	20.96	0.875	6.90

**Table 4:** PSNR and SSIM values were obtained from the validation of pre-trained models under the RS-haze category on the RICE-I dataset.

Model	PSNR	SSIM	psnr×0.3+ssim×0.7
dehazeformer-b	19.13	0.800	6.30
dehazeformer-m	19.21	0.808	6.33
dehazeformer-s	19.10	0.807	6.30
dehazeformer-t	18.61	0.803	6.14

**Table 5:** PSNR and SSIM values were obtained from the validation of pre-trained models under the indoor category on the RICE-II dataset.

Model	PSNR	SSIM	psnr×0.3+ssim×0.7
dehazeformer-b	16.48	0.627	5.38
dehazeformer-m	17.16	0.644	5.60
dehazeformer-s	17.21	0.651	5.62
dehazeformer-t	16.86	0.633	5.50

**Table 6:** PSNR and SSIM values were obtained from the validation of pre-trained models under the outdoor category on the RICE-II dataset.

Model	PSNR	SSIM	psnr×0.3+ssim×0.7
dehazeformer-b	16.97	0.646	5.54
dehazeformer-m	16.59	0.633	5.42
dehazeformer-s	16.74	0.633	5.46
dehazeformer-t	16.82	0.639	5.49

**Table 7:** PSNR and SSIM values were obtained from the validation of pre-trained models under the RESIDE-6K category on the RICE-II dataset.

Model	PSNR	SSIM	psnr×0.3+ssim×0.7
dehazeformer-b	16.25	0.615	5.30
dehazeformer-m	16.36	0.620	5.34
dehazeformer-s	16.44	0.625	5.37
dehazeformer-t	16.33	0.621	5.33

**Table 8:** PSNR and SSIM values were obtained from the validation of pre-trained models under the RS-haze category on the RICE-II dataset.

Model	PSNR	SSIM	psnr×0.3+ssim×0.7
dehazeformer-b	18.99	0.680	6.17
dehazeformer-m	19.00	0.658	6.16
dehazeformer-s	18.74	0.671	6.09
dehazeformer-t	18.74	0.674	6.09

**Table 9:** Models obtained by training the three algorithms, AOD-Net, FFA-Net, and DehazeFormer, on the RICE-I dataset.

Arithmetic	Model
AOD-Net	AOD-train-model-I
FFA-Net	FFA-train-model-I
DehazeFormer	DehazeFormer-train-model-I

**Table 10:** Models obtained by training the three algorithms, AOD-Net, FFA-Net, and DehazeFormer, on the RICE-II dataset.

Arithmetic	Model
AOD-Net	AOD-train-model-II
FFA-Net	FFA-train-model-II
DehazeFormer	DehazeFormer-train-model-II

**Table 11:** Comparison between pre-trained and trained models of the three algorithms, AOD-Net, FFA-Net, and DehazeFormer.

**RICE-I**	**Arithmetic**	**Model**	**PSNR**	**SSIM**	psnr×0.3+ssim×0.7
	AOD-Net	Pretrained models	17.68	0.707	5.799
		AOD-train-model-I	15.80	0.755	5.269
	FFA-Net	Pretrained models	15.30	0.697	5.078
		FFA-train-model-I	14.78	0.453	4.751
	DehazeFormer	Pretrained models	17.70	0.824	5.887
		DehazeFormer-train-model-I	18.13	0.813	6.008
**RICE-II**	**Arithmetic**	**Model**	**PSNR**	**SSIM**	psnr×0.3+ssim×0.7
	AOD-Net	Pretrained models	15.08	0.405	4.808
		AOD-train-model-I	17.15	0.700	5.635
	FFA-Net	Pretrained models	17.92	0.727	5.885
		FFA-train-model-I	14.57	0.291	4.575
	DehazeFormer	Pretrained models	17.30	0.533	5.563
		DehazeFormer-train-model-I	28.09	0.862	9.030

Note: The number highlighted in the table is the calculated maximum value under the algorithm for that row.

**Table 12:** PSNR and SSIM values of each algorithm under the RICE-I dataset.

Methods	PSNR	SSIM	psnr×0.3+ssim×0.7
DCP	16.52	0.798	5.5146
AOD-Net	16.65	0.740	5.513
FFA-Net	20.13	0.775	6.5815
DehazeFormer	25.31	0.902	8.2244

**Table 13:** PSNR and SSIM values of each algorithm under the RICE-II dataset.

Methods	PSNR	SSIM	psnr×0.3+ssim×0.7
DCP	13.78	0.567	4.5309
AOD-Net	19.83	0.795	6.5055
FFA-Net	18.45	0.756	6.0642
DehazeFormer	28.22	0.832	9.0484

**Table 14:** Parameters and FLOPs values for each algorithm and its test model.

Method/Model	Parameters	FLOPs
DehazeFormer-train-model-I	1876.57 Billion	4.633504 × 10^6^
DehazeFormer-train-model-II	939.92 Billion	2.514348 × 10^6^
AOD-Net	1761	2.28851712 × 10^8^
FFA-Net	4,455,913	2.329659047936 × 10^12^

## Data Availability

The datasets used and/or analyzed during the current study, as well as the program code, are available from the corresponding author on reasonable request.
